# Exploring the impact of short daily haemodialysis on muscle strength and bone health in end‐stage kidney disease patients

**DOI:** 10.1002/jcsm.13428

**Published:** 2024-01-25

**Authors:** Fernanda Silveira Tavares, Hugo de Luca Corrêa, Kenneth R. Wilund, Lysleine Alves Deus, Thais Branquinho de Araújo, Carmen Tzanno‐Martins, Vitória Marra da Motta Vilalva Mestrinho, Rafael Lavarini dos Santos, Andrea Lucena Reis, Fernando Honorato Souza, Luiz Roberto de Sousa Ulisses, Helen Souto Siqueira Cardoso, Istênio José Fernandes Pascoal, Valéria Cunha Campos Guimarães, Lucy de Oliveira Gomes, Rodrigo Vanerson Passos Neves, Thiago dos Santos Rosa

**Affiliations:** ^1^ Department of Medicine Catholic University of Brasilia Brasília Brazil; ^2^ Graduate Program of Physical Education Catholic University of Brasilia Brasília Brazil; ^3^ Center for Transplantation Sciences, Department of Surgery Massachusetts General Hospital, Harvard Medical School Boston United States; ^4^ Department of Kinesiology and Community Health University of Illinois Urbana‐Champaign Urbana IL USA; ^5^ Graduate Program of Genomic Sciences and Biotechnology Catholic University of Brasilia Brasilia Brazil; ^6^ Clinical Group Home Dialysis Center and RenalClass São Paulo Brazil; ^7^ NefroClínicas, Premium Nephrology Clinic Brasilia Brazil; ^8^ Centro Brasiliense de Nefrologia e Diálise Brasilia Brazil; ^9^ Graduate Program of Gerontology Catholic University of Brasilia Brasilia Brazil

**Keywords:** Bone mineral density, Conventional haemodialysis, End‐stage kidney disease, Inflammation, Muscle function, Short‐daily haemodialysis

## Abstract

**Background:**

Short‐daily haemodialysis (SDH) has been strongly recommended over conventional haemodialysis (CHD) for end‐stage kidney disease patients, though few studies have directly compared the effects of these two haemodialysis (HD) modalities on clinical variables related to patient's health.

**Methods:**

We conducted a cross‐sectional study in individuals undergoing HD, comparing epidemiological, clinical, metabolic, inflammatory, anthropometric, bone health/metabolism, and skeletal muscle function according to dialysis modality. One‐hundred seventy‐eight patients (20.8% females, 62 ± 2.5 years old), were analysed in this study, 86 (48%) of whom were undergoing CHD versus 92 (51%) who were undergoing SDH.

**Results:**

SDH patients had significantly higher serum albumin levels (3.93 vs. 3.66 g/dL, *P* < 0.0001) and higher Kt/v (2.6 vs. 2.38, *P* < 0.0001). SDH group presented a significantly lower number of erythropoietin‐stimulating agents compared with CHD group (percentage: 53.3 vs. 83.7%, *P* < 0.0001) and had lower levels of serum phosphate (4.9 vs. 5.3 mg/dL, *P* = 0.004) and parathyroid hormone (PTH) (398.4 vs. 480.4 pg/mL, *P* < 0.001) compared with CHD patients. In terms of bone health and metabolism, SDH patients had significantly higher total BMD, femur BMD, lumbar BMD, and femoral neck BMD compared with CHD patients (all *P* < 0.05). SDH patients also had lower anti‐osteogenic and inflammatory biomarkers, including FGF23, sclerostin, TNF, IL‐18, IL‐17a, and C‐reactive peptide (all *P* < 0.05). CHD modality was demonstrated to be a risk factor for low BMD (odds ratio: 4.02; 95% CI: 1.59–10.2, *P* = 0.003). In terms of skeletal muscle function, SDH patients had significantly higher 6‐minute walking test (444.6 vs. 424.9 m, *P* = 0.04) and higher fat‐free mass (52.3 vs. 51.68 kg, *P* = 0.02) compared with CHD patients. Higher fat‐free mass and handgrip strength were associated with a 34% and 23% lower risk of low BMD, respectively. SDH patients had lower levels of the uremic toxin asymmetric dimethyl‐l‐arginine (ADMA) (1.8 vs. 2.07 μM, *P* = 0.002) and fasting blood glucose (132.6 vs. 141.7 mg/dL, *P* < 0.02) than CHD group. SDH patients also displayed higher levels of haemoglobin when compared with CHD group (11.9 vs. 10.2 g/dL, *P* < 0.0001).

**Conclusions:**

The present study improves our understanding of the relationship between dialysis modality and clinical variables that may influence HD patient's health. Grip strength and lean mass were positively correlated with bone mineral density in HD patients regardless of dialysis modality. SDH was associated with better bone mineral density, inflammatory profile, and skeletal muscle function when compared with CHD patients. These findings provide more evidence of the clinical benefits of SDH that should be explored in greater detail.

## Introduction

The kidneys play an important role in regulating signalling pathways related to skeletal muscle and bone health.^1^, ^2^, ^3^ Patients with glomerular filtration rate (GFR) < 15 mL/min are defined as having end stage kidney disease (ESKD) and require renal replacement therapy, which may include transplantation, peritoneal dialysis, or haemodialysis (HD).^3^ Kidney failure is associated with the development and progression of multiple comorbid conditions, including osteoporosis, cachexia, chronic inflammation, iron deficiency, and cardiovascular diseases.^4^, ^5^, ^6^, ^7^, ^8^

Most individuals with ESKD undergo conventional haemodialysis (CHD), which is performed in an outpatient dialysis clinic 2 to 4 days/week for 3 to 6 h/session. However, this treatment schedule was established in the 1970s based on economic and feasibility considerations to maximize the number of HD patients that could be treated, as opposed to any clinical benefits.^9^ There is now increasing evidence that the prognosis of HD patients can be improved by increasing the number or duration of HD sessions.^10^, ^11^, ^12^, ^13^, ^14^ In this regard, the application of short‐daily in‐center HD (SDH) (2–3 h, five or more sessions per week) may provide promising advantages compared with CHD as it appears to attenuate HD‐related adverse effects.^10^, ^11^, ^12^, ^14^, ^15^, ^16^

SHD appears to reduce chronic inflammation and left ventricular hypertrophy and is associated with better phosphorus control when compared with CHD.^17^ Furthermore, moving from CHD to SDH reduced chronic volume overload, reducing the risk of mortality in this population.^17^ SDH also is considered more physiologically similar to normal renal function, which may help explain the plethora of benefits in HD patients.^10^, ^11^, ^12^, ^14^, ^16^, ^17^ However, due to the persistent bone mineral disorders, cachexia, and related comorbidities,^1^, ^2^, ^3^, ^6^, ^7^, ^8^, ^11^ there is a growing interest in examining whether patients undergoing SDH could have improved bone and muscle characteristics compared with CHD patients.

The first aim of the present study was to identify clinical variables that may be differentially affected by SDH and CHD. The second aim was to determine if the positive association between skeletal muscle health (handgrip strength and fat‐free mass) and bone mineral density is affected by HD modality.

## Methods

This is a cross‐sectional study comparing epidemiological, clinical, metabolic, inflammatory, anthropometric, bone health/metabolism, and skeletal muscle function in patients undergoing maintenance haemodialysis. The study includes patients from six different private clinical centres, consisting of three centres from the same company and three centres from different companies. It is important to note that all of these clinics provide care to patients who are subsidized by the government. Among the ESKD patients included in the study, some are undergoing short daily haemodialysis (SHD), while others are receiving conventional haemodialysis (CHD). Two hundred seventy‐one ESKD patients were enrolled. The following inclusion criteria were applied: ≥18 years old; undergoing chronic HD for at least the past 3 months; no complications from unstable metabolic medical diseases as outlined by a nephrologist; no apparent cardiovascular complications (such as heart failure, severe arrhythmia, angina or cerebrovascular disease). Patients were excluded if they presented any of the following: severe decompensated metabolic disorders; stroke in the last 6 months; adequacy dialysis (Kt/V) < 1.2; history of access to arteriovenous fistula in the previous 3 months; and/or presence of autoimmune disease(s). After applying exclusion criteria, 178 patients remained in the study. Eighty‐six (48%) were undergoing CHD, while 92 (51%) were performing SDH (Figure S1), leading to a sample power of 91% based on an effect size of 0.5 and an alpha of 5% (calculated by G*power version 3.1.). This study was approved by the local Ethics Committee (23007319.0.0000.0029).

### Anthropometric measures and bone mineral density

All subjects were weighed on a mechanical scale (Filizola®, São Paulo, Brazil), and height was measured with a stadiometer built into the scale (precision: 0.5 cm). Body composition and BMD were measured using a Prodigy Advance Plus (LUNAR, Corp/General Electric; Madison, Wisconsin, USA) dual‐energy X‐ray absorptiometry (DXA) according to previously specified procedures.^18^ All measurements were carried out by the same experienced researcher.

### Muscle strength and function analysis

Muscle strength was evaluated by handgrip isometric dynamometer Jamar TM® (Sammons Preston, Illinois, USA). Throughout these measurements, the same observer conducted the assessments while maintaining blinding to the participants' conditions. Patients adopted the sitting position with their arms beside the trunk. The elbow, hip, and knee joints were kept at 90° and the wrist in the pronated position. All patients performed an average of three attempts in the contralateral arm of the arteriovenous fistula, with 2 min rest between attempts. In clinical practice, the contralateral arm is typically the dominant arm. The greatest attempt was used as the value of HGS. The technical description of the test execution was described by Correa et al.^7^

For the study of functional capacity, the timed up and‐go test (TUG) and 6‐min walk test (6MWT) were used. Before performing both tests, the procedures were fully explained to patients with subsequent familiarization. For TUG, three attempts were timed with 60 s of rest between them. The best performance was recorded and used for the present analyses; all these procedures were described by Gadelha et al.^19^ In the 6MWT protocol, participants were instructed to wear comfortable clothes and shoes and not to exercise in the 12 h prior to the test. We emphasize that for the execution of this physical test and the others, the participants were authorized by health professionals such as cardiologists, nephrologists, and clinical physiologists. The test was performed in the corridors of the haemodialysis clinics. All patients received standardized verbal instructions for performing the test.^20^

### Blood collection and storage

Blood collections were performed in the morning before the haemodialysis session in all patients. Blood was collected in vacutainer tubes containing EDTA (1.00 mg/mL) (an anticoagulant). Blood was then centrifuged (15 min, 3.000× *g*, 4°C) to obtain plasma and was stored in vacutainer tubes at −80°C until analysis according to Neves et al.^18^

### Biochemical assays

The following biochemical variables were measured according to standard methods at the routine clinical laboratory: parathyroid hormone (PTH), calcium (Ca), haemoglobin, glycated haemoglobin (HbA1c), fasting blood glucose (FBG) lipid profile, C‐reactive peptide (CRP), and phosphorus (P). Serum levels of Klotho, fibroblast growth factor 23 (FGF23) (IBL Co., Ltd, Japan, and Immutopics Inc., USA), and sclerostin (Biomedica Gruppe, Vienna, Austria) were determined in duplicate using specific human enzyme‐linked immunosorbent assay (ELISA) kits. The detectable limit for Klotho, FGF23, and sclerostin was 6.15 pg/mL, 10 pg/mL, and 3.2 pmol/L, respectively. Both assays had intra‐ and inter assay CVs <10%. Vitamin D (1,25 (OH)_2_ vitD) was measured by the radioimmunoassay method (DiaSorin Diagnosis, Saluggia, Italy).

The systemic levels of tumour necrosis factor‐alpha (TNF‐α), interleukin 6 (IL‐6), IL‐10, and IL‐18 were measured in triplicate by ELISA kits from R&D Systems (Minneapolis, MN) according to manufacturer instructions. Serum IL‐17a concentration was determined by ELISA method (Mini ELISA Development Kit, 900‐M21; PeproTech Inc., Rocky Hill, NJ). The detectable limit for TNF‐α, IL‐6, IL‐10, IL‐18, and IL‐17a was 10, 18, 0.2, <1, and 10 pg·mL^−1^, respectively. The overall intra‐ and inter assay CVs values for inflammatory markers were in a range of 4% to 10%.

NO bioavailability (assessed by NO_2_^−^ levels) were analysed as previously described by Correa et al.^20^ ADMA concentration was determined by ELISA, the intra‐ and inter‐assay CV were ≤8% and ≤12%, respectively (Human ADMA ELISA Kit, MyBioSource, San Diego, USA).

### Statistical analysis

Descriptive characteristics are presented as means and standard deviations unless otherwise noted. The Shapiro–Wilk test and Levene were used to verify data distribution nature and homogeneity, respectively. Data were explored using hierarchical cluster analysis, which identified four possible cluster for the data (Clusters 1, 2, 3, and 4). An independent *χ* ^2^ test was used to compare the proportion of the dialysis type on each cluster. Kruskal–Wallis followed by Dunn's post‐hoc was applied to compare bone mineral density and handgrip strength for each cluster formed. Principal component analysis was performed to identify the top 15 variables that contributes to the total variance. With these top 15 variables, we performed a structural equation model using the lavaan package^21^ to identify the crosstalk between handgrip strength and bone mineral density with the other variables. Two models were created according to the variable nature (model 1: physiology and model 2: molecular). The model adequacy criteria were adopted according with the following parameters: comparative fit index (CFI) approximately to 0.9, root mean square error of approximation (RMSEA) below 0.8, and standardized root mean square residual (SRMR). In the present study, the model presented CFI = 0.86; RMSEA = 0.12; SRMR = 0.06.

A Student's *t* test or Mann–Whitney was used to compare continuous variables among the dialysis groups (CHD and SHD). A multinomial regression was performed to identify associations between dialysis type, HGS, fat‐free mass, and BMD. Data were tested for multicollinearity and the Hausman–McFadden test was applied to verify the independence of irrelevant alternatives. Odds ratio (95% confidence intervals [95%]) was calculated based on high mineral density (cut points defined for BMD were based on terciles: low <1.093 cm^2^; reduced >1.093 and <1.168 cm^2^; high >1.168.) and SDH as reference categories. Given the diverse characteristics observed in CKD patients, utilizing terciles is a method that effectively captures the specificity of our sample. Graph visualization was performed according to the Heru Wiryanto [https://rpubs.com/heruwiryanto/multinom‐reg]. All analysis was performed using the software R and RStudio version 4.1.3.

## Results

Out of 178 subjects, 86 (48%) were undergoing CHD, and 92 (51%) were receiving SDH. On average, patients in our study received 12 h of haemodialysis per week. It is worth noting that CHD sessions were typically divided into approximately six sessions of 2 h each, whereas SDH sessions consisted of three sessions lasting 4 h each. There was no change in the modality for any of the patients during the study. Heparin was used as an anticoagulant, with doses ranging from 100 to 220 units per kilogram, and an average dose of 150 units per kilogram was administered. For patients with catheters, an additional 1 mL of heparin was administered at the end of the haemodialysis session. The main causes of renal disease in our cohort were diabetes (42.7%), glomerulonephritis (4.5%), hypertension (48.9%), and polycystic kidney disease (3.9%). Among the patients in the study, 70% (*n* = 124) had previously used erythropoietin at some point during their haemodialysis (HD) treatment. During the study period, 19.66% (*n* = 35) of the patients were actively using erythropoietin, with 11.23% (*n* = 20) in the conventional HD group and 8.4% (*n* = 15) in the daily HD group. Most patients received erythropoietin dosages of 50 IU/kg three times a week, occasionally increasing to 150 IU/kg. A total of 22.47% (*n* = 40) of the patients (29 from the conventional HD group and 11 from the daily HD group) were prescribed vitamin D at a dose of 50,000 IU once a week for 4 weeks, coinciding with the study period. Furthermore, 40.44% (*n* = 72) of the patients (43 from the conventional HD group and 29 from the daily HD group) were using Sevelamer, a phosphate binder, at a dosage of 800 mg two to three times a day before their main meals.

Patients were clustered into four groups: Clusters 1, 2, 3, and 4, according to the similarity between individuals (Figure S2A). The proportion of SDH and CHD differed between clusters (*χ* ^2^ = 17.4, *df* = 3, *P* < 0.001, Cramer V = 0.31, Figure S2B). PCA indicated the top 15 variables that explained the most variance (Figure S2C,D). Figure S3 demonstrates the path diagram between HGS and bone mineral density with the top 15 variables indicated by PCA analysis. Cluster 1 presented lower BMD when compared with Cluster 2 (1.09 ± 0.1 vs. 1.18 ± 0.11; *P* < 0.001) and Cluster 4 (1.09 ± 0.1 vs. 1.16 ± 0.05; *P* < 0.001). Similar results were observed for HGS, which was lower on Cluster 1 when compared with Cluster 2 (16.9 ± 3.48 vs. 24.33 ± 4.901; *P* < 0.001) and Cluster 4 (16.9 ± 3.48 vs. 22.69 ± 4.93 *P* < 0.001).

CHD patients displayed a higher albumin, and number of erythropoiesis agents' administration when compared with SDH. Furthermore, SDH patients presented a higher week Kt/v than CHD, See Table 1. A better bone mineral health was found for SDH group when compared with CHD due to higher values of Total femur, L3‐L4, and 1,25 (OH)_2_ vitD. Furthermore, SDH group also presented lower values of FGF‐23, sclerostin, PTH, phosphorus, Ca × PO_4_^3−^, and pro‐inflammatory cytokines (*P* < 0.05). See Table 2.

**Table 1:** Demographic characteristics

Variables	Total cohort (n = 178)	CHD (n = 86)	SDH (n = 92)	*P* value
Body mass (kg)	75.15 ± 5.47	74.88 ± 5.7	75.4 ± 5.26	0.525
Age (years)	62.11 ± 2.55	62.5 ± 2.41	61.75 ± 2.63	0.05
SBP (mmHg)	143.51 ± 8.72	144.76 ± 8.76	142.35 ± 8.58	0.07
DBP (mmHg)	105.8 ± 9.07	106.86 ± 9.09	104.71 ± 8.98	0.11
Time haemodialysis (months)	60.78 ± 3.62	60.51 ± 3.61	61.02 ± 3.64	0.350
Albumin (g/dL)	3.56 ± 0.49	3.16 ± 0.33	3.93 ± 0.27	<0.0001
Week Kt/v	2.49 ± 0.31	2.38 ± 0.26	2.6 ± 0.32	<0.0001
Sex (women/men)	37 (20.8) /141 (79.2)	19 (22.1) /67 (77.9)	18 (19.6) /74 (80.4)	0.678
Catheter	21 (11.8)	10 (11.6)	11 (12)	0.968
Beta‐blockers	49 (27.5)	22 (25.6)	27 (29.3)	0.574
Calcium channel blocker	30 (16.9)	14 (16.3)	16 (17.4)	0.843
Smoke (yes)	45 (25.3)	21 (24.4)	24 (26.1)	0.798
Erythropoiesis agents	121 (68)	72 (83.7)	49 (53.3)	<0.0001
Phosphate binder	70 (39.3)	38 (44.2)	32 (34.8)	0.199

Data presented as mean ± standard deviation and *n* (%). Independent *t* test or Mann–Whitney was performed to compare groups.

CHD, conventional haemodialysis; SDH, short‐daily haemodialysis.

**Table 2:** Bone mineral density and bone‐renal biomarkers according to the dialysis modality

Variables	CHD (n = 86)	SDH (n = 92)	Mean difference	95% CI	*P* value
Total BMD (cm^2^)	1.11 ± 0.11	1.16 ± 0.12	−0.03	(−0.06–0.004)	0.003
Total femur (cm^2^)	0.88 ± 0.1	0.95 ± 0.12	−0.07	(−0.1 – −0.04)	<0.0001
Femoral neck (cm^2^)	0.82 ± 0.1	0.84 ± 0.1	−0.02	(−0.05–0.01)	0.029
L3–L4 (cm^2^)	1.07 ± 0.14	1.11 ± 0.13	−0.04	(−0.08–0.001)	0.04
Klotho (pg/mL)	141.16 ± 63.66	155.28 ± 49.72	−14.12	(−30.96–2.71)	0.1
FGF‐23 (UR/mL)	718.55 ± 171.31	646.25 ± 194.68	72.3	(18.13–126.46)	0.009
Klotho/FGF‐23	6.22 ± 3.08	4.64 ± 2.22	1.58	0.78–2.38	<0.0001
Sclerostin (pg/mL)	2.02 ± 0.65	1.71 ± 0.61	0.31	(0.12–0.5)	0.001
PTH (pg/mL)	480.43 ± 170.8	398.43 ± 197.17	82.00	(27.26–136.73)	0.004
1.25 (OH)_2_ vitD (pg/mL)	47.64 ± 17.71	57.86 ± 21.5	−10.22	(−16.03 – −4.41)	<0.0001
Calcium (mg/dL)	9.39 ± 1.76	9.02 ± 1.69	0.37	(−0.14–0.88)	0.153
Phosphorus (mg/dL)	5.27 ± 0.93	4.86 ± 0.92	0.41	(0.13–0.68)	0.004
Ca × PO_4_^3−^	50.35 ± 16.17	45.2 ± 16	2.41	(0.38–9.91)	0.03
TNF (pg/mL)	31.1 ± 5.9	27.7 ± 5.1	4.57	(3.04–6.1)	<0.0001
IL‐18 (pg/mL)	1,358 ± 343	1211.22 ± 296	170.28	(76.48–264.08)	0.003
IL‐10 (pg/mL)	6.69 ± 3.19	6.55 ± 3.04	0.14	(−0.78–1.06)	0.765
IL‐17a (pg/mL)	30.09 ± 4.55	28.55 ± 4.75	1.53	(0.16–2.91)	0.029
IL‐6 (pg/mL)	28.98 ± 5.2	27.81 ± 6.64	1.17	(−0.58–2.93)	0.189
CRP (pg/mL)	1.23 ± 0.58	1.03 ± 0.57	0.2	(0.03–0.37)	0.02

Data expressed as mean and standard deviation. Independent *t* test or Mann–Whitney was performed to compare groups.

BMD, bone mineral density; CHD, conventional haemodialysis; CRP, C‐reactive protein; FGF‐23, fibroblast growth factor 23; IL, interleukin; PTH, parathormones; SDH, short‐daily haemodialysis; TNF, tumour necrosis factor.

Patients from the SDH group presented higher values of fat‐free mass, 6‐min walking test performance, and haemoglobin, while patients from CHD group presented higher values of interdialytic weight gain, fasting blood glucose, and ADMA (*P* < 0.05). Data are described in Table 3.

**Table 3:** Anthropometric functional performance metabolic profile and nitric oxide bioavailability according to dialysis modality

Variables	CHD (n = 86)	SDH (n = 92)	Mean difference	95% CI	P value
Fat mass (kg)	23.3 ± 5.62	23.09 ± 5.28	0.21	(−1.4–1.82)	0.80
Fat‐free mass (kg)	51.68 ± 1.58	52.31 ± 1.91	−0.63	(−1.14 – −0.11)	0.02
Handgrip strength (kgf)	20.02 ± 5.62	21.78 ± 6.31	−1.76	(−3.53–0.01)	0.05
Interdialytic weight gain (kg)	4.12 ± 0.98	2.67 ± 0.92	1.46	(1.17–1.74)	<0.0001
Timed up and go test (s)	10.24 ± 2.26	10.06 ± 1.91	0.18	(−0.44–0.8)	0.57
6‐min walking test (m)	424.92 ± 59.8	444.61 ± 67.25	−19.69	(−38.56 – −0.82)	0.04
Fasting blood glucose (mg/dL)	141.74 ± 24.62	132.57 ± 26.64	9.18	(1.58–16.78)	0.02
Haemoglobin (g/dL)	10.24 ± 1.52	11.98 ± 1.23	−1.74	(−2.15 – −1.33)	<0.0001
HbA1c (%)	6.39 ± 1.32	6.26 ± 1.19	0.13	(−0.24–0.51)	0.47
Total cholesterol (mg/dL)	187.69 ± 23.87	183.98 ± 23.04	3.71	(−3.23–10.65)	0.29
Triglycerides (mg/dL)	241.28 ± 39.56	231.35 ± 35.31	9.93	(−1.15–21.01)	0.08
HDLc (mg/dL)	29.92 ± 8.87	29.66 ± 6.8	0.26	(−2.1–2.61)	0.83
LDLc (mg/dL)	109.51 ± 25.1	108.05 ± 25.29	1.47	(−5.99–8.93)	0.70
NO_2_^−^ (μM)	42.2 ± 16.04	45.94 ± 14.43	−3.74	(−8.25–0.77)	0.10
ADMA (μM)	2.07 ± 0.54	1.78 ± 0.64	0.29	(0.11–0.46)	0.002

Data expressed as mean and standard deviation. Independent *t* test was performed to compare groups.

ADMA, asymmetric dimethyl‐l‐arginine; CHD, conventional haemodialysis; HbA1c, glycated haemoglobin; HDLc, high density lipoprotein; LDLc, low density lipoprotein; NO_2_^−^, nitrite; SDH, short‐daily haemodialysis.

Patients with higher fat‐free mass and handgrip strength had a 34% and 23% reduced chance, respectively, of presenting with low BMD. Furthermore, CHD modality was associated with low BMD (odds ratio: 4.02; 95% CI: 1.59–10.2; *P* = 0.003). Handgrip strength also was associated with increased odds of having reduced BMD (odds ratio: 0.89; 95% CI: 0.83–0.96; *P* = 0.003). The pseudo *R* ^2^ of the model was 0.39. See Table 4.

**Table 4:** Multinomial logistic regression

Characteristics	Odds ratio	95% CI	*P*‐value
Reduced BMD			
Fat‐free mass	0.81	0.64–1.03	0.086
Handgrip strength	0.89	0.83–0.96	0.003
HD type			
SDH	Ref	Ref	Ref
CHD	1.85	0.83–4.07	0.13
Low BMD			
Fat‐free mass	0.66	0.49–0.88	0.005
Handgrip strength	0.77	0.70–0.84	<0.0001
HD type			
SDH	Ref	Ref	Ref
CHD	4.02	1.59–10.2	0.003

Reference: high BMD (>1.168 cm^2^). Pseudo *R* ^2^ = 39%. Cut points defined for BMD were based on terciles: low <1.093 cm^2^; reduced >1.093 and <1.168 cm^2^; high >1.168.

BMD, bone mineral density; CHD, conventional haemodialysis; CI, confidence interval; HD, haemodialysis; SDH, short‐daily haemodialysis.

**Figure 1:**
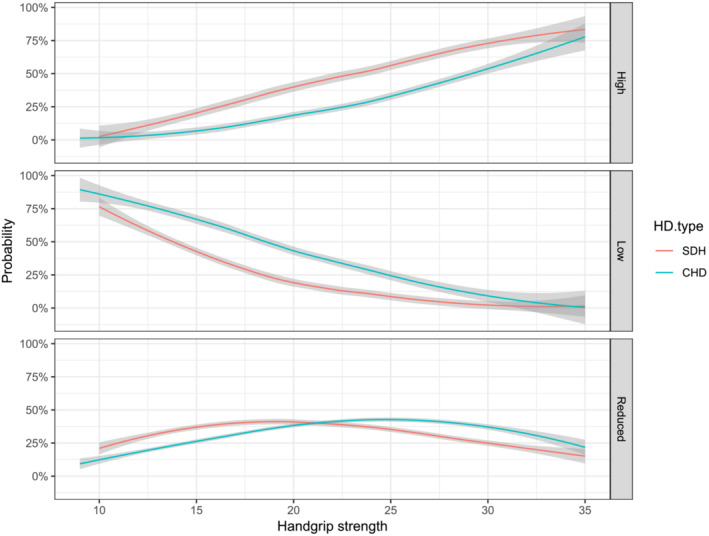
Probability of presenting with a high, low, or medium BMD according to HD modality. Pseudo *R* ^2^ = 39%. Cut‐off points defined for BMD were based on terciles: Low <1.093 cm^2^; reduced >1.093 and <1.168 cm^2^, and high >1.168. CHD, conventional haemodialysis; HD, haemodialysis; SDH, short‐daily haemodialysis.

Figure 1 illustrates the individual probability to present a high, medium, or low BMD according to handgrip strength and dialysis modality. Notably, higher values of handgrip strength are associated with a higher BMD. In general, patients from SDH group are likely to present with higher BMD. However, according to the increase of the values of handgrip strength, the effect of dialysis became similar between CHD and SDH. Therefore, patients with higher strength seems to present higher BMD regardless of the dialysis type.

## Discussion

This study demonstrated that SDH was associated with better bone mineral density/metabolism, muscle mass/function, inflammatory profile, glycaemic status, haemoglobin levels, and markers of endothelial health (ADMA) when compared with CHD. These observations may be important for the care of HD patients since these variables are known risk factors for cardiovascular events, hospitalization, and mortality in HD patients, regardless of dialysis type.^22^, ^23^ We also found that the bone mineral density was higher in those with increased handgrip strength, demonstrating a possible protective effect of muscle strength on bone density in this population (Figure 1). Therefore, a valuable finding of this study was that muscle mass and strength may counteract chronic kidney disease‐related decrease on bone mineral density. This phenomenon should be tested in further longitudinal studies aimed to verify the causal relation of skeletal muscle function and bone health.

A growing body of evidence suggests that fracture susceptibility is increased as CKD progresses.^5^, ^6^, ^24^ Slouma et al.^5^ demonstrated that lower BMD might be associated with advanced age and higher concentrations of PTH in HD patients. This same study demonstrated that FGF23 was increased in patients with lumbar osteoporosis. Taken together, patients undergoing maintenance HD are at high risk of osteopenia/osteoporosis, leading to a higher incidence of bone fractures and hospitalizations. Therefore, a key finding of the present study was that SDH patients presented with a better femur and lumbar mineral density, increased pro‐osteogenic molecules (Klotho/FGF23 ratio and vitamin D), and lower levels of molecules related to bone loss, including FGF23, sclerostin, PTH, phosphorus, and Ca × PO_4_^3−^ (Table 2). This finding suggests that SDH may be a feasible strategy to prevent osteopenia and osteoporosis.

Osteoporosis seems related to several endocrine and metabolic pathways that are strongly influenced by inflammation.^25^ Proinflammatory cytokines, including TNFα, IL6, IL18, IL17, and CRP appear to activate osteoclasts and osteoblasts, leading to bone metabolism disorders.^23^, ^25^ Inflammation is a common feature of chronic kidney disease, which *per se* could be a significant factor for bone loss in this population.^23^ In this regard, lower levels of TNFα, IL18, IL17, and CRP presented by SDH patients in this study partially explains their better bone density and metabolic profile.

Adipose tissue is an important regulator of inflammation,^26^ with elevated fat mass being associated with higher inflammation.^27^ We expected that SDH patients would present with lower fat mass than CHD, but no differences were found between groups (Table 3). In this regard, we believe that the lower values of pro‐inflammatory cytokines in the SDH group may be partially explained by their increased muscle mass and function, but not fat mass. A recent study suggests that elevated muscle mass is associated with increased production of myokines, which can improve clinical outcomes, including cognition, bone formation, endothelial function, inflammation, and metabolic profile.^28^ These findings corroborate with the present study as SDH patients presented with better muscle mass and function, along with better fasting blood glucose, haemoglobin, and ADMA. ADMA, an endogenous inhibitor of the nitric oxide synthases, leads to several adverse outcomes, including oxidative stress, inflammation, and endothelial diseases.^29^, ^30^ Lower concentrations of ADMA in SDH patients may help explain the protective effect of this dialysis modality in ESKD patients.

Based on the theoretical assumption that muscle strength and mass might be associated with better bone mineral density, we performed a multinomial regression with these three variables according to the dialysis modality. This analysis helped clarify why handgrip strength did not differ between groups (Table 3). Based on the analysis, there is a greater probability (approximately 75%) in presenting with high BMD in patients with stronger handgrip strength regardless of dialysis type. This relationship was greatest in the weakest patients, which presented lower probabilities (approximately 5%) of having high BMD (*Figure* *2*). This finding provides evidence of a potential relationship between handgrip strength and BMD, reinforcing the concept of a so‐called bone muscle crosstalk.^31^, ^32^ A study in the general US population demonstrated a possible association between HGS with BMD.^33^ Our data suggests this association may also be present in HD patients, though this finding needs to be confirmed with additional research.

This study presents some limitations that should be considered: (i) The cross‐sectional design did not allow cause‐effect inferences. (ii) We did not control for nutritional status, physical activity levels, socioeconomic status or other factors which may have influenced the results. (iii) While our study categorized BMD into three groups based on terciles to provide a general overview of bone health and metabolism, we acknowledge the importance of considering age and sex differences in BMD at specific anatomical sites. To address this concern, future studies should plan to expand theis analysis to examine BMD at specific sites and incorporate *Z*‐scores into the assessments, as recommended by previous investigation.^34^ This refined approach will provide a more comprehensive understanding of how age and sex may influence BMD variations at different anatomical sites within our study cohort, enhancing the depth and relevance of our findings. We recognize these potential limitations and encourage further studies to examine these topics.

The present study adds new evidence regarding the protective effects of SDH in ESKD patients. In general, SDH patients presented with a better disease prognosis compared with CHD patients. Our data also suggest that patients with higher grip strength have improved bone mineral density and metabolic parameters regardless of dialysis modality. More research is needed to determine if exercise or pharmacological interventions to improve muscle strength will also enhance bone health in patients undergoing CHD and SDH. Herein, we provided novel insights on how HD patients could benefit from undergoing SDH. Furthermore, multinomial analysis suggested a protective role of muscle strength and SDH on bone mineral density.

## Conflict of interest

None.

## Funding

This study was financed in part by the Conselho Nacional de Desenvolvimento Científico e Tecnológico (CNPq) and Coordenação de Aperfeiçoamento de Pessoal de Nível Superior—Brasil (CAPES)—Finance code 001. This work was funded by the Fundação de Apoio à Pesquisa do Distrito Federal (FAP/DF) with grants from: demanda espontânea – Grant number 09/2022.

## Supporting information


**Figure S1.** **Flow diagram of patients' inclusion.**
**Figure S2.** Exploratory analysis of 178 haemodialysis patients. Hierarchical cluster dendrogram (**1A**), chi‐squared graph (**1B**), biplot PCA variables (**1C**), top15 contributor variables (**1D**). CHD: conventional haemodialysis; SDH: short‐daily haemodialysis; PCA: principal components analysis; HGS: handgrip strength; TUG: timed‐up and go test, PTH: parathormone; BMD: total bone mineral density; FGF: fibroblast growth factor; 6MWT: 6‐minute walking test.
**Figure S3.** Path diagram with top15 variables presented on PCA. Coefficients between HGS and BMD is 0.56. PCA: principal components analysis; HGS: handgrip strength; TUG: timed‐up and go test, PTH: parathormone; BMD: total bone mineral density; FGF: fibroblast growth factor; 6MWT: 6‐minute walking test.

**Data S1.** Supporting Information.

## Data Availability

All data and code used for analysis are available in Data S1 or at 10.6084/m9.figshare.24082182.
