# Supplementary material for: Exploring the impact of short daily haemodialysis on muscle strength and bone health in end‐stage kidney disease patients
Source: J Cachexia Sarcopenia Muscle. 2024 Jan 25;15(2):718–25. doi: 10.1002/jcsm.13428 (PMC10995248; doi:10.1002/jcsm.13428)
Supplement: Supplementary file 1 — Figure S1. Flow diagram of patients' inclusion. Figure S2. Exploratory analysis of 178 haemodialysis patients. Hierarchical cluster dendrogram (1A), chi‐squared graph (1B), biplot PCA variables (1C), top15 contributor variables (1D). CHD: conventional haemodialysis; SDH: short‐daily haemodialysis; PCA: principal components analysis; HGS: handgrip strength; TUG: timed‐up and go test, PTH: parathormone; BMD: total bone mineral density; FGF: fibroblast growth factor; 6MWT: 6‐minute walking test. Figure S3. Path diagram with top15 variables presented on PCA. Coefficients between HGS and BMD is 0.56. PCA: principal components analysis; HGS: handgrip strength; TUG: timed‐up and go test, PTH: parathormone; BMD: total bone mineral density; FGF: fibroblast growth factor; 6MWT: 6‐minute walking test. [file JCSM-15-718-s001.docx]

Supplementary material


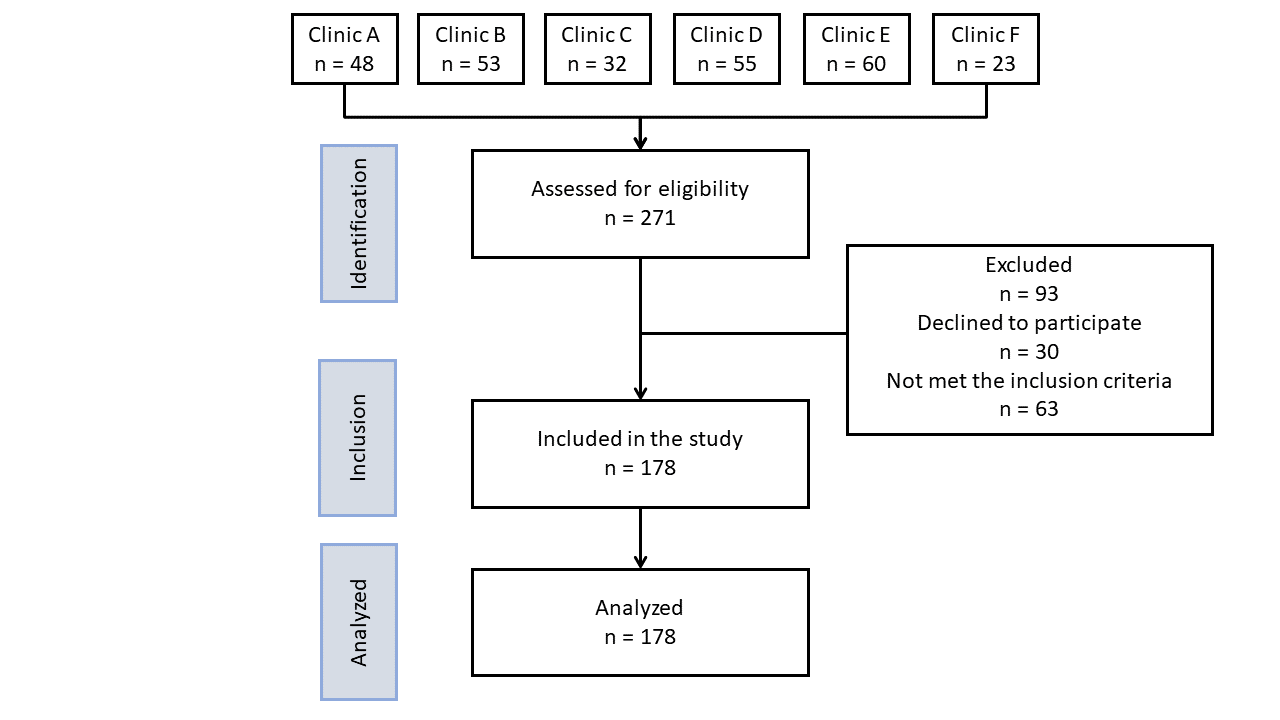


**Supplementary Figure 1. Flow diagram of patients’ inclusion.**


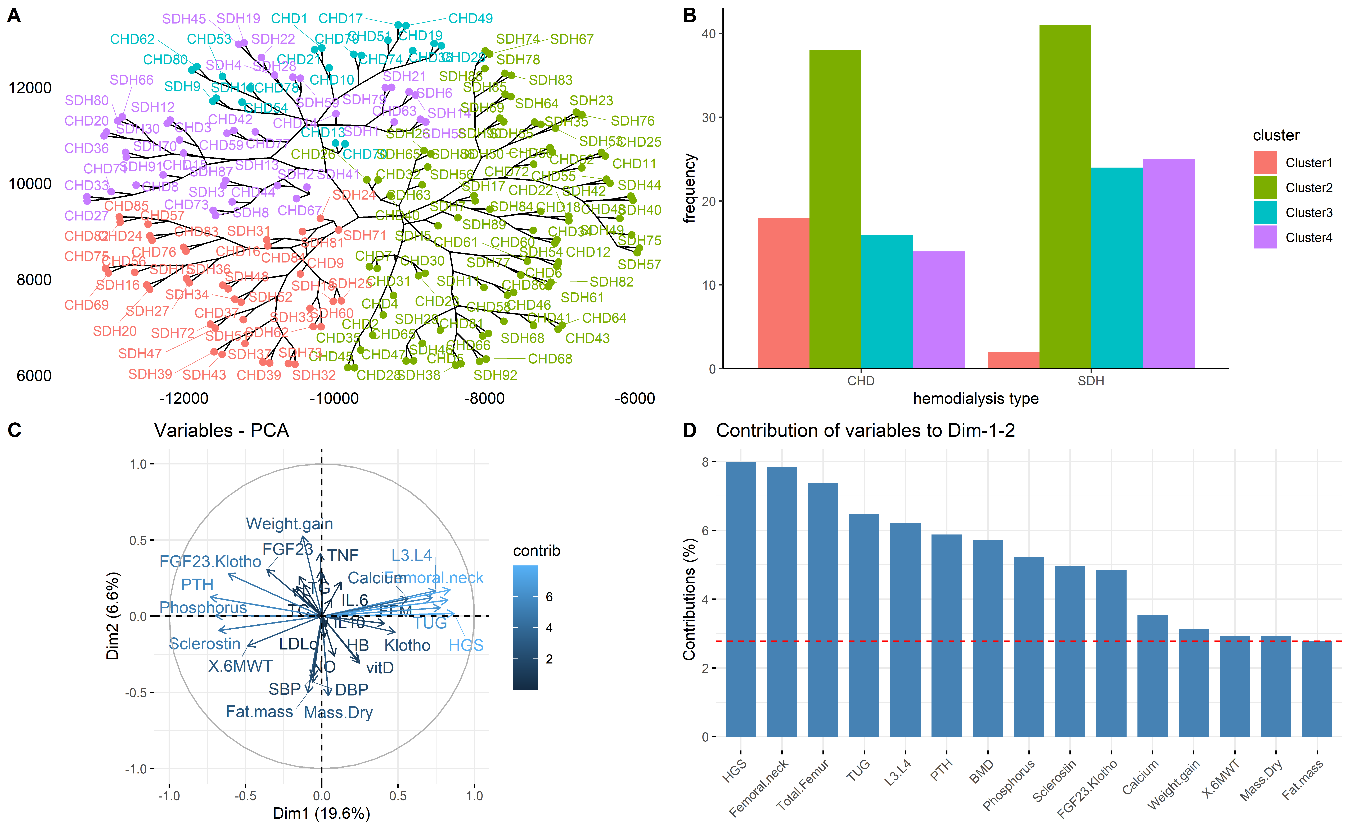


**Supplementary Figure 2.** Exploratory analysis of 178 hemodialysis patients. Hierarchical cluster dendrogram (**1A**), chi-squared graph (**1B**), biplot PCA variables (**1C**), top15 contributor variables (**1D**). CHD: conventional hemodialysis; SDH: short-daily hemodialysis; PCA: principal components analysis; HGS: handgrip strength; TUG: timed-up and go test, PTH: parathormone; BMD: total bone mineral density; FGF: fibroblast growth factor; 6MWT: 6-minute walking test.


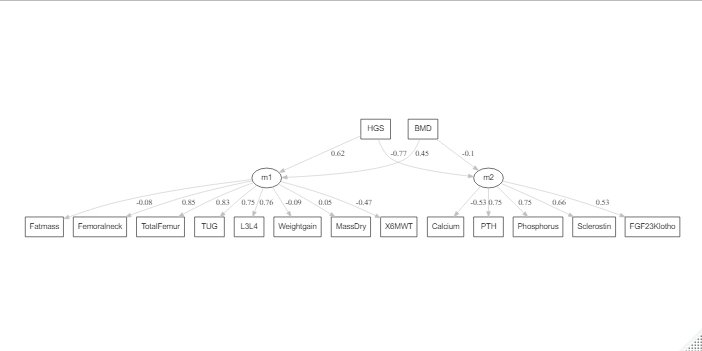


**Supplementary Figure 3.** Path diagram with top15 variables presented on PCA. Coefficients between HGS and BMD is 0.56. PCA: principal components analysis; HGS: handgrip strength; TUG: timed-up and go test, PTH: parathormone; BMD: total bone mineral density; FGF: fibroblast growth factor; 6MWT: 6-minute walking test.
